# Supplementary material for: Content-rich biological network constructed by mining PubMed abstracts
Source: BMC Bioinformatics. 2004 Oct 8;5:147. doi: 10.1186/1471-2105-5-147 (PMC528731; doi:10.1186/1471-2105-5-147)
Supplement: Additional File 2 — The original results of the above study (non-essential files are deleted to keep the file size under the limit set by BMC bioinformatics). [file 1471-2105-5-147-S2.bz2 › chilibotAdditionalFile2/dip10/322ID10576729E2443/html/ATPG_ATPE.html]

 


 **ATPG** and **ATPE** 
  
Found 8 abstracts in PubMed, retrieved 8.  
 

 What does Google say? 
 PDF only 
| .edu only 

---

**Interactive relationship** (e.g. stimulation, inhibition, etc)

**Non-interactive relationship** (e.g. studied together, co-existance, homology, etc.)

- The affinities of binding of 30 S ribosomal subunits showed the relationship  **atpE**  greater than atpB greater than  **atpG** .  Ref: 2693739 J Mol Biol, 1989
- Five genes,  **atpE** , atpF, atpH, atpA and  **atpG** , were shown to be translationally coupled to various degrees of tightness.  Ref: 1830358 Mol Microbiol, 1991
- Moreover, phytanic acid activates brown adipocyte differentiation long term exposure of brown preadipocytes to phytanic acid promoted the acquisition of the brown adipocyte morphology and caused a co ordinate induction of the mRNAs for gene markers of brown adipocyte differentiation, such as UCP1, adipocyte lipid binding protein  [ **ATPE** ]  aP2, lipoprotein lipase, the glucose transporter GLUT4 or subunit II  [ **ATPG** ]  of cytochrome c oxidase.  Ref: 11829740 Biochem J, 2002
- The control of the atp genes expression rates has been shown to be exercised primarily at the level of translational initiation, but how is this achieved in molecular terms ? In order to study the interactions of 30 S ribosomal subunits with specifically the translational initiation regions TIRs of atpB,  **atpE**  and  **atpG** , restriction fragments bearing these TIRs were excised from the atp operon and cloned into an SP6 promoter transcription vector.  Ref: 2693739 J Mol Biol, 1989
- The C. thermoaceticum atp operon contained nine genes arranged in the order atpI i, atpB a,  **atpE**  c, atpF b, atpH delta, atpA alpha,  **atpG**  gamma, atpD beta, and atpC epsilon.  Ref: 9171425 J Bacteriol, 1997
- Eight genes atpI, atpB,  **atpE**  1,  **atpE**  2,  **atpE**  3, atpF, atpH, and atpA upstream of and contiguous with the previously described genes  **atpG** , atpD, and atpC were cloned from chromosomal DNA of Acetobacterium woodii.  Ref: 10567365 J Biol Chem, 1999
